# Dysfunction of γδ T cells in pediatric chronic active Epstein-Barr virus infection

**DOI:** 10.1186/s13052-024-01783-x

**Published:** 2024-10-12

**Authors:** Junhong Ai, Haijuan Xiao, Linlin Zhang, Honghao Ma, Dong Wang, Dilara Dilmurat, Ran Wang, Zhengde Xie

**Affiliations:** 1 Laboratory of Infection and Virology, Beijing Pediatric Research Institute, Beijing Children’s Hospital, Capital Medical University, National Center for Children’s Health, Beijing, 100045 China; 2 Research Unit of Critical Infection in Children, 2019RU016, Chinese Academy of Medical Sciences, Beijing, 100045 China; 3 Department of Infectious Diseases, Beijing Children’s Hospital, Capital Medical University, National Center for Children’s Health, Beijing, 100045 China; 4 Medical Oncology Department, Pediatric Oncology Center, Beijing Children’s Hospital, Capital Medical University, National Center for Children’s Health, Beijing, 100045 China; 5 Hematology Center, Beijing Children’s Hospital, Capital Medical University, National Center for Children’s Health, Beijing, 100045 China

**Keywords:** Epstein-Barr virus, Chronic active Epstein-Barr virus infection, γδ T cells, Pediatric, Dysfunction

## Abstract

Chronic active Epstein-Barr virus infection (CAEBV) is a progressive and life-threatening disease characterized by persistent or recurrent EBV activation. It has been reported that, γδ T cells, a type of cytotoxic lymphocyte, play a critical role in restricting EBV. However, the functional status of γδ T cells in pediatric CAEBV patients has not yet been described. In this study, flow cytometry analysis was conducted to explore the cytokine production capacity of γδ T cells in CAEBV patients. A diminished frequency of γδ T cells and decreased expression of cytolytic molecule granzyme B were found in CAEBV patients, suggesting a dysfunction in the immune regulatory function of γδ T cells in this disease.

## Introduction

Epstein-Barr virus (EBV) is a ubiquitous human gamma herpesvirus, infecting over 90% of the global population. Typically, individuals acquire EBV during early childhood, and this initial infection usually manifests asymptomatic. However, delayed acquisition of EBV may lead to infectious mononucleosis (IM), characterized by acute but generally benign and self-limiting symptoms [1]. In rare cases, individuals infected with EBV may develop chronic active EBV infection (CAEBV), a condition marked by persistent or recurrent IM-like symptoms for more than 3 months. CAEBV poses a significant risk to health and can potentially become life-threatening [2].

Cytotoxic lymphocytes, such as CD8^+^ T cells, NK cells, and γδ T cells, play a crucial role in the immune control during EBV infection [3]. Among these, γδ T cells represent a unique subset of cytotoxic lymphocytes known for their potent innate immune responses, capable of swiftly recognizing antigens in an MHC-independent manner. Studies have demonstrated an elevation the frequency of γδ T cells in the peripheral blood of individuals with IM resulting from acute EBV infection [4]. Moreover, a significant portion of γδ T cell in IM patients exhibit positivity for the activation marker CD38, underscoring their antiviral function during acute EBV infection [4].

γδ T cells primarily exert their function through the production of various cytokines, which facilitate cytotoxicity against target cells. Studies have indicated that during chronic viral infections, such as human immunodeficiency virus infection, chronic hepatitis B virus infection, and hepatitis C virus infection, the capacity of γδ T cells to produce cytokines and execute cytotoxic functions becomes compromised [5]. However, the status of γδ T cells, both in terms of frequency and functionality, in patients with CAEBV remains poorly understood due to the sustained viral activity and prolonged nature of the disease. Therefore, the aim of the study is to investigated the cytokine production capacity of γδ T cells in children with CAEBV using flow cytometry analysis.

## Methods

A total of 10 pediatric patients diagnosed with CAEBV (Table 1) and 18 age-matched healthy carriers (HC) of EBV (Table S1) during routine physical examinations were enrolled in this study at the Beijing Children’s Hospital, Capital Medical University. Peripheral blood samples were collected from all participants.

**Table 1:** Clinical information of chronic active EBV infection (CAEBV) patients

Patient No	Gender	Age (years)	EBV load in plasma (copies/mL)	EBV load in PBMC (copies/mL)	Major lymphocyte subsets of EBV infection
CA01	M	10.08	9.44 × 10^3^	1.26 × 10^6^	NK
CA02	F	7.25	6.33 × 10^2^	1.17 × 10^7^	T
CA03	M	6.00	9.58 × 10^2^	7.96 × 10^6^	Not determined
CA04	F	11.08	8.05 × 10^2^	2.15 × 10^6^	T
CA05	F	13.00	9.61 × 10^2^	1.08 × 10^7^	T
CA06	F	2.00	< 500	1.72 × 10^6^	T
CA07	F	2.00	< 500	5.56 × 10^6^	T
CA08	F	5.00	< 500	1.46 × 10^5^	Not determined
CA09	F	15.50	< 500	7.28 × 10^3^	NK
CA10	M	9.75	< 500	1.95 × 10^6^	NK

*CA* CAEBV, *M* Male, *F* Female, *T* T cells, *NK* Natural killer cells

In humans, γδ T cells can be classified into Vδ1, Vδ2 and Vδ3 subtypes, with Vγ9Vδ2 T cells comprising 60–90% of peripheral blood γδ T cells [6]. Previous studies have shown that EBV-positive Daudi cells can vigorously activate Vγ9Vδ2 T cells *in vitro* [7]. Therefore, in this study, peripheral blood mononuclear cells (PBMCs) from both HC and patients with CAEBV patients were isolated using density gradient centrifugation. Subsequently, these PBMCs were subjected to stimulation with Daudi cells to conduct functional assays targeting γδ T cells. In brief, 5 × 10^5^ PBMCs were cultured either alone or in the presence of Daudi cells at an effector-to-target cell ratio of 10:1. The cells were cultured for 24 h in RPMI 1640 medium with 10% fetal bovine serum and recombinant human IL-2 (25 ng/ml) (R&D Systems). For cytokine detection, GolgiStop protein transport inhibitor (BD Biosciences) was added to the cultures for the last 5 h. Flow cytometry analysis was then performed to measure the frequency of γδ T cell expressing granzyme B, CD107a, perforin, IFN-γ, and TNF-α. The flow cytometry analysis strategy is illustrated in Fig. S1.

Data were analyzed using GraphPad Prism Software and are presented as mean ± SEM.* P* values were calculated using two-tailed unpaired Student’s* t* tests, with *P* values of < 0.05 considered statistically significant.

## Results and discussion

**Fig. 1:**
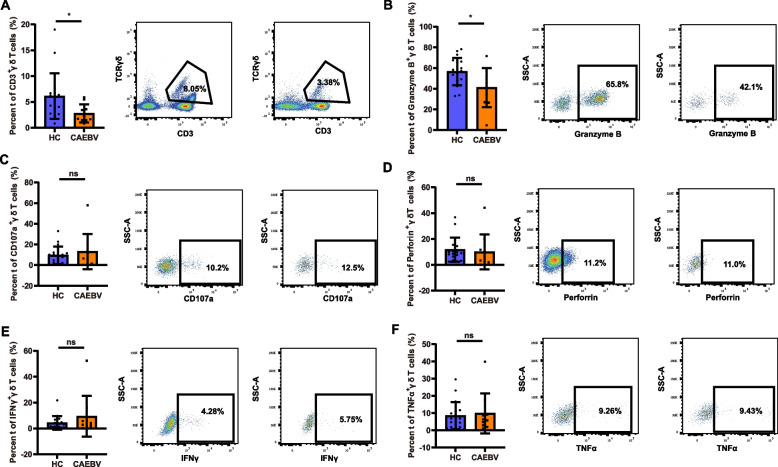
Expression of cytolytic molecules in circulating γδ T cells in CAEBV. PBMCs were isolated from the peripheral blood of patients with CAEBV (*n* = 10) and EBV healthy carriers (HC) (*n* = 18). The PBMCs were incubated alone or in the presence of Daudi cells and then analyzed using a BD LSRFortessa flow cytometer. **A** The proportion of γδ T cells in PBMCs. **B**-**F** The expression levels of granzyme B, CD107a, perforin, IFN-γ, and TNF-α in γδ T cells, respectively. Each point in the histogram represents a patient's data, and mean values ± SEM are also provided. ns, no statistical difference. ^*^, *P* < 0.05

The results revealed a significant decrease in the percentage of γδ T cells in CAEBV patients compared to HC (*P* < 0.05, Fig. 1A). Previous studies have indicated a negative correlation between EBV reactivation and the recovery of γδ T cells following hematopoietic stem cell transplantation, underscoring the pivotal role of γδ T cells in restricting EBV reactivation [8]. Hence, the observed reduction in γδ T cells in CAEBV may associated with the persistent activation of EBV during the development of the disease. The functionality of γδ T cells hinges on their ability to release cytotoxic effector molecules such as granzyme B, perforin, and IFN-γ, which aid in eliminating infected, stressed, and transformed cells. To understand the role of γδ T cells in CAEBV, we investigated their capacity to produce these cytotoxic effector molecules. Our findings, illustrated in Fig. 1B, demonstrated a significant reduction in granzyme B levels in γδ T cells from CAEBV patients compared to those in HC (*P* < 0.05). Conversely, the expression levels of CD107a, perforin, IFN-γ and TNF-α exhibited no discernible between CAEBV patients and HC (*P* > 0.05, Fig. 1C-F). CAEBV can be divided into T cell type and NK cell type according to the lymphocyte mainly infected by the virus. In this study, we found that in CAEBV patients, whether they have the T cell type or the NK cell type, the frequency of γδ T cells decreases and granzyme B secretion is reduced.

Previously, the γδ T cell function was found to be impaired in EBV-associated nasopharyngeal carcinoma, characterized by diminished IFN-γ and TNF-α production and weakened killing capacity against nasopharyngeal carcinoma cell lines *in vitro* [9, 10]. Although CAEBV is not been explicitly classified as malignant disease, it exhibits malignant characteristics due to clonal proliferation of EBV-infected cells and has the potential to progress to hematological malignancies, such as T-cell lymphoma [11]. The reduced expression of the cytolytic molecule granzyme B in CAEBV patients suggests an insufficient ability of γδ T cell to eradicate EBV-infected clonal expansion cells, although definitive conclusions necessitate further cytotoxicity experiments. CAEBV patients may have cytokine imbalances, such as elevated levels of immunosuppressive cytokines like IL-10, which can inhibit the activation and proliferation of γδ T cells [12], and prolonged chronic infection may lead to γδ T cell exhaustion [13], thereby reducing granzyme B secretion [14].

γδ T cells have dual antiviral and antitumor effects. Studies have shown that adoptive immunotherapy based on γδ T cells has effectively controlled EBV-induced B lymphoproliferative disease after transplantation *in vitro* and in humanized mouse models [15]. Therefore, immunotherapy based on γδ T cells may be a potential effective strategy for the treatment of CAEBV in the future.

In summary, the diminished frequency of γδ T cells and the decreased expression of cytolytic molecule granzyme B in CAEBV signify a dysfunction in the immune regulatory function of γδ T cells in this disease.

## Data Availability

The datasets used and/or analysed during the current study are available from the corresponding author on reasonable request.

## Abbreviations

EBV: Epstein-Barr virus
IM: Infectious mononucleosis
CAEBV: Chronic active Epstein-Barr virus infection
HC: Healthy carriers
PBMC: Peripheral blood mononuclear cells
